# Does Lysosomial Acid Lipase Reduction Play a Role in Adult Non-Alcoholic Fatty Liver Disease?

**DOI:** 10.3390/ijms161226085

**Published:** 2015-11-25

**Authors:** Francesco Baratta, Daniele Pastori, Licia Polimeni, Giulia Tozzi, Francesco Violi, Francesco Angelico, Maria Del Ben

**Affiliations:** 1 Department of Internal Medicine and Medical Specialities and Department of Anatomical, Histological, Forensic Medicine and Orthopedics Sciences-Sapienza University, Rome 00185, Italy; francesco.baratta@uniroma1.it (F.B.); daniele.pastori@uniroma1.it (D.P.); licia.polimeni@uniroma1.it (L.P.); 2 Unit for Neuromuscular and Neurodegenerative Diseases, Children’s Hospital and Research Institute “Bambino Gesù”, Rome 00165, Italy; giulia.tozzi@opbg.net; 3 Department of Internal Medicine and Medical Specialities, Sapienza University, Rome 00185, Italy; francesco.violi@uniroma1.it (F.V.); maria.delben@uniroma1.it (M.D.B.); 4 Department of Public Health and Infectious Diseases, Sapienza University, Policlinico Umberto I, I Clinica Medica, Viale del Policlinico 155, Rome 00161, Italy

**Keywords:** lysosomial acid lipase, non-alcoholic fatty liver disease, Wolman Disease, cholesterol ester storage disease, hypercholesterolemia

## Abstract

Lysosomal Acid Lipase (LAL) is a key enzyme involved in lipid metabolism, responsible for hydrolysing the cholesteryl esters and triglycerides. Wolman Disease represents the early onset phenotype of LAL deficiency rapidly leading to death. Cholesterol Ester Storage Disease is a late onset phenotype that occurs with fatty liver, elevated aminotransferase levels, hepatomegaly and dyslipidaemia, the latter characterized by elevated LDL-C and low HDL-C. The natural history and the clinical manifestations of the LAL deficiency in adults are not well defined, and the diagnosis is often incidental. LAL deficiency has been suggested as an under-recognized cause of dyslipidaemia and fatty liver. Therefore, LAL activity may be reduced also in non-obese patients presenting non-alcoholic fatty liver disease (NAFLD), unexplained persistently elevated liver transaminases or with elevation in LDL cholesterol. In these patients, it could be indicated to test LAL activity. So far, very few studies have been performed to assess LAL activity in representative samples of normal subjects or patients with NAFLD. Moreover, no large study has been carried out in adult subjects with NAFLD or cryptogenic cirrhosis.

## 1. Introduction

Non-alcoholic fatty liver disease (NAFLD) is a spectrum of disorders characterized by excessive hepatic fat accumulation that occurs in individuals in the absence of significant alcohol consumption or chronic viral infection. NAFLD is the most common hepatic disease involving a growing number of people worldwide. In the general population, the prevalence of NAFLD is about 20%–30%, and reaches 70%–90% in obese or diabetic patients [1]. The early stage of NAFLD is represented by simple steatosis, where the main histologic finding is the presence of fatty liver; in some cases simple steatosis my evolve in non-alcoholic steatohepatitis (NASH), where steatosis is associated with hepatocellular injury and inflammation with or without fibrosis.

Traditionally, NAFLD has been interpreted as a benign condition; however, more recent evidence suggests that NAFLD may progress to advanced liver disease such as cirrhosis, hepatocellular carcinoma, and end stage hepatic failure [2].

NAFLD is the result of many different pathogenic mechanisms which cause lipid accumulation into hepatocytes [3], increased oxidative stress, pro-inflammatory changes [4], and eventually fibrosis in a subset of individuals.

The mechanisms underlying the evolution of simple steatosis to NASH and/or liver cirrhosis are not yet clarified, and the progression of NAFLD is not predictable.

Nowadays, NAFLD is a major cause of cryptogenic cirrhosis, whose prevalence has increased over the last years especially in patients with a history of obesity and type 2 diabetes. NAFLD is the third most common indication for liver transplantation in the United States and is projected to eventually overtake the hepatitis C virus and alcoholic liver disease and to become the main cause of liver transplants [5].

## 2. Non-Alcoholic Fatty Liver Disease and Cardiovascular Disease

Prospective studies suggested that, in patients with NAFLD, cardiovascular disease (CVD) is the first cause of death [6]. Thus, atherosclerosis is the primary cause of morbidity for these subjects, and many of them will be suffering from CVD before the development of liver-related complications [7].

The association between NAFLD and CV risk has been largely investigated [8], but a definite explanation has not been provided. Among the proposed mechanisms, it has been suggested that NAFLD, especially in its more advanced forms, might act itself as a stimulus for the release of pro-atherogenic factors contributing actively to the onset of CVD [9].

The association of steatosis with different pro-atherogenic conditions is another plausible reason accounting for an increased CV risk [10]. Thus, patients with NAFLD disclosed systemic signs of atherosclerosis, such as increased carotid intima-media thickness and endothelial dysfunction [11].

Common metabolic disorders, such as dyslipidaemia, type 2 diabetes [12] and central obesity, have been associated with both simple liver steatosis and progressive NASH.

Besides, it has been also suggested that fatty liver can be considered an hepatic consequence of the insulin resistance related to the metabolic syndrome (MetS) [13,14], which is a highly pro-atherogenic condition that involves approximately 20% of the non-diabetic population in the western countries meeting the ATPIII diagnostic criteria [15].

Insulin resistance is a paramount pathophysiological moment in the MetS, and according to the “two hit” hypothesis, is also considered to play a central role in the first stage of fatty liver infiltration [16]. However, whether MetS with insulin resistance promotes fatty liver or whether NAFLD itself induces chronic hyperinsulinemia by impaired insulin degradation, is still under debate. The current opinion is that there is a strong bidirectional association between NAFLD and MetS [9].

However, not all NAFLD cases could be explained by insulin resistance; in fact, not all subjects with MetS will develop NAFLD and not all subjects with NAFLD have MetS or will develop it.

### PNPLA3 and Non-Metabolic NAFLD

Patatin-like phospholipase domain-containing protein 3 (PNPLA3) is a gene encoding a lipase enzyme expressed in adipocytes. The mutation of PNPLA3, such as the PNPLA3 MM genotype, showed to be strongly associated with the presence of NAFLD and NASH [17]. Patients with PNPLA3 MM genotype do not show classical metabolic features commonly described in NAFLD patients with wild type genotype. In fact, normal peripheral and hepatic insulin sensitivity has been described in NAFLD patients with PNLPA3 mutation [18,19].

In addition, NAFLD patients with PNPLA3 mutation showed a lower CV risk compared to “metabolic” NAFLD patients, questioning as to whether NAFLD represents an independent CV risk factor.

## 3. Clinical Presentations of Genetic LAL Deficiency

Lysosomal Acid Lipase (LAL) deficiency is a rare autosomal recessive genetic disease characterized by the accumulation of cholesteryl esters (CE) and triglycerides in many tissues, caused by mutations of the gene encoding LAL, namely *LIPA* gene [20]. The most common *LIPA* gene mutation is the E8SJM variant, and its frequency is 0.0025 in the general population; this translates into a carrier frequency of about one in 200 in Western countries [21].

LAL deficiency is a heterogeneous disease and two main different phenotypes may be present; the Wolman Disease represents the early onset of LAL deficiency and manifests itself during the first six months of life, and it is rapidly fatal for the patient. Babies with LAL deficiency show growth retardation associated with malabsorption, hepatosplenomegaly, severe liver dysfunction, rapidly progressive anaemia and multi-organ failure. Adrenal calcification is the pathognomonic sign of Wolman Disease. The survival beyond one year of age is very rare.

Cholesterol Ester Storage Disease (CESD) is a late onset phenotype that occurs with fatty liver, elevated aminotransferase levels, hepatomegaly and dyslipidaemia characterized by elevated low-density lipoprotein cholesterol (LDL-C) and low high-density lipoprotein cholesterol (HDL-C) with or without triglyceride elevation. CESD may manifest in infancy, childhood or adulthood, and it remains often unrecognized since symptoms can overlap with other conditions. Patients have a more variable age of clinical presentation, ranging from five years to 44 years or over, and milder clinical courses [22].

The natural history and the clinical manifestations of the disease in children and adults are less well defined and the diagnosis is often incidental. Lipid abnormalities are common, and patients may present early signs of systemic atherosclerosis. Moreover, hepatomegaly and microvescicular steatosis with liver cell damage and splenomegaly are common features of the disease [23].

Clinical phenotype and the severity of LAL deficiency depend on the magnitude of the residual enzymatic activity. Therefore, finding steatosis and NASH in non-obese patients with lipid abnormalities may help in differentiating LAL deficiency from other metabolic causes of NAFLD such as MetS, type 2 diabetes, hypertriglyceridemia and central obesity [24].

## 4. Liver Histology in LAL Deficiency

The relationship between LAL deficiency and histological liver alterations was investigated only in subjects with CESD or Wolman Disease.

Based on available data, all patients with *LIPA* gene disorders have liver steatosis. Often, the differential diagnosis with other causes of fatty liver can be difficult and a definitive diagnosis can be done only by histological analysis of liver biopsy specimens.

In paraffin fixed specimen, the main feature is represented by a pervasive and homogeneous microvescicolar steatosis, although this aspect is not specific for CESD [23]. Conversely, in unfixed frozen samples, the finding of cholesterol ester crystals, using polarized light, is a distinctive feature of CESD [25].

Recently, Hůlková H. *et al.* [25] provided a new immunohistochemistry method to better identify CESD, in both paraffin-fixed and frozen biopsy specimen. The presence of luminal cathepsin D and membrane lysosomal markers namely lysosomal-associated membrane protein 1 and 2, and lysosomal integral membrane protein 2 around the lipid vacuoles, confirms the intra-lysosomal lipid accumulation. Moreover, the presence of macrophage with intracellular ceroid accumulation is another common histological finding in patients with CESD. The presence of this specific feature, namely ceroid induction, localized in lysosomes from macrophage, but not in those from hepatocytes, supports the diagnosis of CESD.

## 5. The Role of LAL in Lipid Metabolism

LAL is a key enzyme involved in intracellular lipid metabolism and trafficking; it is responsible for the intra-lysosomal hydrolysis of LDL CE and triglycerides into free cholesterol and free fatty acids [26]. Therefore, the reduction of LAL activity determines intra-lysosomal lipid accumulation and a consecutive reduction of free cholesterol in cytosol [27]. This can promote an increase of the activity of the sterol regulatory element-binding proteins (SREBPs), leading to increased lipogenesis, cholesterol biosynthesis and VLDL production. At the same time, there is also a reduction of the expression of liver X receptors (LXRs) leading to reduced efflux of cholesterol and HDL production. Therefore, abnormalities in serum lipids are induced.

The main evidence of lipid serum alterations, in LAL activity deficiency, derives from studies performed in patients with homozygous genetic disorders for *LIPA* gene.

The most common lipoprotein alterations in patients with homozygous LAL deficiency are type IIa (high LDL-C with normal triglycerides) and type IIb dyslipidaemias (high LDL-C and triglycerides), combined with low HDL-C. In these patients, dyslipidaemia has been associated with accelerated atherosclerosis. Therefore, in the presence of a type IIa dyslipidaemia, the differential diagnosis with heterozygous familial hypercholesterolemia (HeFH) is very important but not always easy to perform. The presence of family history for premature CVD and/or for hypercholesterolemia may contribute to make FH diagnosis. By contrast, in the absence of diagnostic criteria for HeFH, diagnosis of LAL deficiency should be suspected.

Further studies were carried out in heterozygous patients for *LIPA* gene mutation. A recent review on patients with different LIPA mutations [23], reported an increase of total and LDL-C, and most patients had a severe LDL-C elevation (>200 mg/dL). In 65 patients, HDL cholesterol was determined, and, in 57 of those, it was found to be reduced. Premature atherosclerosis was also documented in some patients. Based on the above study, it appears that the occurrence of lipid alterations and of accelerated atherosclerosis is similar in patients with LAL deficiency due to homozygous and heterozygous mutation of *LIPA* gene. LAL deficiency should more often be considered in dyslipidemic patients with combined hyperlipidemia and low HDL-C.

Only one study explored lipid data in patients with non-genetic LAL activity reduction. Authors reported a moderate elevation of total and LDL-C in NAFLD patients with lower LAL activity. No differences were reported in HDL-C and triglycerides.

All the above data suggest a negative correlation between LAL activity and total and LDL-C elevation.

## 6. The Role of LAL in Atherosclerosis

It has been recently hypothesized that changes in LAL activity could contribute to the atherosclerotic process. The formation and accumulation of foam cells within wall arteries is a key pathophysiological moment in the formation of atherosclerotic plaque [28].

Foam cells derived from oxidation of lipid products, mostly in the form of CE, that cannot be metabolized upon LDL receptor pathway and are recognized and removed by scavenger receptors expressed on macrophages and smooth muscular cells, leading to accumulation of cholesterol in these cells [27]. Thus, CE are physiologically hydrolysed in the lysosomes by LAL to generate free cholesterol, which, after being re-esterified in the endoplasmic reticulum, can form cytosolic lipid droplets. The accumulation of free cholesterol in lysosomes during the atherosclerotic process could inhibit LAL activity, causing accumulation of CE in cells. LAL is also present within the extracellular space of atherosclerotic intima [29].

Physiopathology findings have been confirmed by interventional studies on mice with recombinant human LAL, in which a reversal of atherosclerotic lesions have been observed [27].

## 7. Who Should Be Tested for LAL Activity?

LAL activity reduction should always be suspected in non-obese patients presenting with NAFLD and/or cryptogenic cirrhosis, unexplained persistently elevated liver transaminases or with elevation in LDL-C and decreased HDL-C (Table 1). An accurate anamnesis is necessary to exclude potential causes contributing to fatty liver, such as viral causes, alcohol abuse or the presence of familial hypercholesterolemia [24].

In these patients, it could be indicated to test LAL activity, using the dried blood spot (DBS) test. The DBS is a simple test used to determine LAL activity by comparing total lipase activity to lipase activity in the presence of a highly specific inhibitor (Lalistat 2) of LAL. It allows the differentiation of healthy subjects from affected individuals. All patients with LAL reduction (≤0.40 nmol/spot/h) detected by DBS should perform genetic tests to detect LAL gene mutations [30].

**Table 1:** Clinical suspicion of lysosomal acid lipase (LAL) reduction.

Who Should Be Tested for LAL Activity?
Patients with unexplained:
•Liver Dysfunction (≥1 of the following)
Persistent elevation of ALT
Presence of hepatomegaly
Hepatic steatosis
AND/OR
•Dislipidemia (≥1 of the following)
High LDL-C (≥160 mg/dL–4.1 mmol/L)
Low HDL-C (≤40 mg/dL–1.0 mmol/L in males; ≤50 mg/dL–1.3 mmol/L in females)

## 8. Current Research Status on LAL Activity and NAFLD

Very few studies have been performed so far to assess LAL activity in representative samples of normal adult subjects or patients with NAFLD. Moreover, no large study has been carried out in adult subjects with NAFLD, and prevalence of *LIPA* gene mutation in this setting is unknown. Only one study investigated the clinical phenotype of patients with heterozygous mutations for *LIPA* genes. However, this study was focused only on lipid panel results and did not show data about hepatic condition or about other biochemical values [21].

*In vitro*, it has been demonstrated that several factors may modulate LAL activity [31]. In particular, enhanced LAL activity was associated with eicosanoids, gonadotropins and glucagon, and reduced activity was correlated with Lp(a), LDL remnants and oxidized LDL concentrations*.*

We recently reported, for the first time, reduced blood LAL activity in adult patients with NAFLD [32]. LAL activity was significantly reduced in 240 patients with NAFLD, as compared to 100 adult subjects [0.78 (0.61–1.01) *vs.* 1.15 (0.94–1.72) nmol/spot/h, *p <* 0.001]. NAFLD patients with LAL activity below median had higher values of serum total cholesterol (*p <* 0.05) and LDL-C (*p <* 0.05), and increased serum liver enzymes (ALT, *p <* 0.001; AST, *p <* 0.01; GGT, *p <* 0.01). We also observed a progressive decrease of LAL activity from patients with simple steatosis [0.84 (0.62–1.08) nmol/spot/h, *p <* 0.001 *vs.* HS] to those with biopsy-proven NASH [0.67 (0.51–0.77) nmol/spot/h, *p <* 0.001 *vs.* HS; *p <* 0.001, among groups].

However, at present, there are no data on certain epigenetic modulation of LAL activity *in vivo* models. Thus, studies are needed to better clarify mechanisms of epigenetic modulation of LAL activity and their potential role as therapeutic targets. For example, we do not know if an intervention on modifiable cardio-metabolic risk factors typically associated with NAFLD, such as metabolic syndrome, overweight, increased oxidative stress, may have a role in modulating LAL activity.

In addition, it is not known if the improvement in LAL activity may translate into a reduction of fatty liver content in adult NAFLD patients.

## 9. Future Directions

Altogether these data indicated that modifications in LAL activity are associated with dyslipidaemia and liver dysfunction [21]. In fact, both serum lipoprotein alterations and NAFLD are common and share many possible pathophysiological mechanisms. Moreover, it is not surprising that LAL activity reduction could be also an unrecognized contributing factor in the development and progression of NAFLD to cryptogenic cirrhosis.

Therefore, the identification of clinical and metabolic risk factors, especially those modifiable, which are able to modulate LAL activity, may have important clinical implications for the management of patients with NAFLD. Moreover, future research should also address epigenetic modulation of LAL activity and also take into consideration the effect of drug treatments. This would be particularly important to better understand the contribution of LAL in the complex scenario of NAFLD.

Recently, Burton BK *et al.* reported an impressive reduction of hepatic fat content as assessed by means of magnetic resonance imaging in patients with severe LAL deficiency treated for 20 weeks with enzyme replacement therapy with Sebelipase alfa [33]. These findings were paralleled by improvement in serum liver enzymes and lipid levels. The study was carried out in subjects with confirmed enzyme activity-based diagnosis performed by dried blood spots using the inhibitor Lalistat 2. Almost 50% of patients had bridging fibrosis at liver biopsy and 31% had cirrhosis.

**Figure 1:**
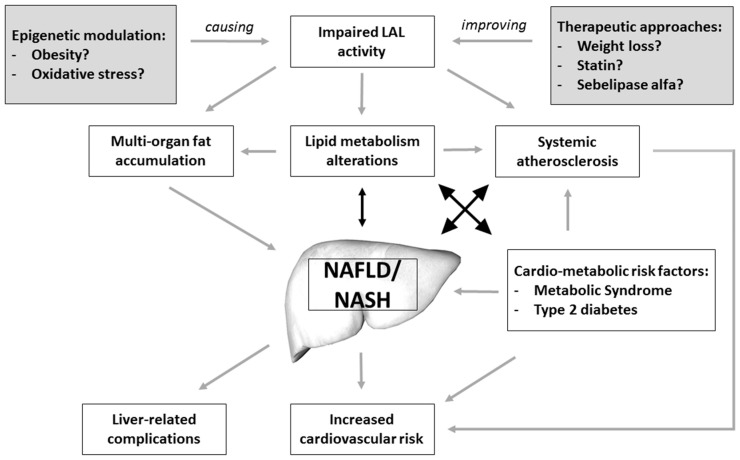
Putative mechanisms linking impaired LAL activity and NAFLD/NASH.

These findings, together with those showing low LAL activity in patients with NAFLD and NASH [32], suggest a strong association between impaired LAL activity and fatty liver pathogenesis and progression. Thus, LAL activity seems to be linked to NAFLD through several mechanisms including lipid metabolism alterations, intra-hepatic fat accumulation and pro-atherosclerotic functions (Figure 1).

Finally, we speculate that LAL activity reduction may become a possible new target for the treatment of NAFLD. In fact, enzyme-replacement therapy may soon be available. This treatment will be indicated for patients with more severe, genetic LAL deficiency, where treatment will be lifesaving. However, in a recent clinical trial lead on CESD patients, treatment with sebelipase was associated with a significant reduction in fatty liver content in almost all treated patients [33]. Based on this evidence, we may speculate that, in the future, enzyme-replacement therapy could be also indicated for less severe LAL deficiency, especially in patients with more advanced forms of NAFLD, such as those with NASH or cryptogenic cirrhosis. Therefore, we believe that it is important to test NAFLD patients for LAL activity to identify a subgroup of patients at higher risk for liver disease progression.
